# Circular RNAs: Emerging players in the pathogenesis of keloid

**DOI:** 10.3389/fphys.2022.1008364

**Published:** 2022-11-09

**Authors:** Xin Yu, Xueqing Zhu, Linfeng Li, Guangcheng Gao

**Affiliations:** Department of Dermatology, Beijing Friendship Hospital, Capital Medical University, Beijing, China

**Keywords:** circular RNAs, circRNAs, keloid, miRNA, mRNA

## Abstract

Circular RNAs (circRNAs) are a new type of non-coding RNAs originating from precursor messenger RNAs. Recent research has confirmed that circRNAs play a significant role in various biological and pathological processes, including cell viability, migration, and apoptosis. Emerging studies have demonstrated that the deregulated circRNA–miRNA–mRNA interaction network plays a key role in the development of many diseases. Increasing evidence has highlighted the role of ncRNAs (mainly miRNAs and lncRNAs) in the pathogenesis of keloids. Recently, several publications also indicated that circRNAs contribute to keloid development. The discovery of circRNAs changed the current understanding of the biology of keloids It is crucial to elucidate a circRNA–miRNA–mRNA network to understand the pathological mechanism of keloids. In the present review, we summarize the aberrant expression of regulatory roles of circRNAs in keloids. We discuss the potential clinical application of circRNAs in the diagnosis and treatment of keloids.

## Introduction

Keloids are pathological scars caused by skin injury and irritation. These are benign skin tumors characterized by hyperproliferation of fibroblasts and excessive deposition of collagen fibers (Limandjaja et al., 2020). Keloids can extend beyond the area of injury and invade the adjacent normal skin (Tan et al., 2019). Keloids significantly reduce people’s life quality by causing esthetic deformity, pruritus, hyperesthesia, and pain (Olaitan, 2009). Conventional treatment ways, consisting of surgical excision, cryotherapy, topical steroids, and laser therapy, remain unsatisfactory, and relapse is common (Park et al., 2014; Berman et al., 2017; Ekstein et al., 2021). It is urgent to understand the mechanism underlying keloid formation for better treatment approaches.

Circular RNAs (circRNAs) are a new type of non-coding RNAs originating from precursor messenger RNAs. Different from linear RNA, circRNAs are single-stranded RNAs that form a closed loop. CircRNAs lack 5′ and 3’ ends, which makes them more stable (Jeck et al., 2013; Kristensen et al., 2019). Since circRNAs were first discovered in plant viroids in 1976, thousands of circRNAs across species have been identified (Sanger et al., 1976; Vo et al., 2019). CircRNAs were considered the byproduct of wrong splicing for a long time. Recent research has confirmed that circRNAs play a significant role in various biological and pathological processes, including cell viability, migration, and apoptosis (Memczak et al., 2013; Di Timoteo et al., 2020). CircRNAs exert their biological functions at the post-transcriptional level, including in transcription and splicing, interfering with miRNA activities or signaling pathways, and serving as translation templates (Chen, 2020). In particular, circRNAs contain various binding sites for miRNAs and absorb miRNAs like a sponge, serving as competitive endogenous RNAs (ceRNAs) or miRNA sponges (Memczak et al., 2013). Emerging studies have demonstrated that the deregulated circRNA–miRNA–mRNA interaction network plays a key role in the development of many diseases (Liang et al., 2020; Sakshi et al., 2021).

Increasing evidence has highlighted the role of ncRNAs (mainly miRNAs and lncRNAs) in the pathogenesis of keloids (Babalola et al., 2013). Recently, several publications also indicated that circRNAs contribute to keloid development. The discovery of circRNAs changed the current understanding of the biology of keloids. It is crucial to elucidate a circRNA–miRNA–mRNA network to understand the pathological mechanism of keloids. In the present review, we summarize the aberrant expression of regulatory roles of circRNAs in keloids. We discuss the potential clinical application of circRNAs in the diagnosis and treatment of keloid.

## The etiology of keloids

Genetic, epigenetic, and environmental factors are crucial in molecular pathogenesis underlying keloid formation (He et al., 2017). The inheritance pattern of keloids may follow autosomal dominance with incomplete penetrance. In addition, keloids tend to be polygenic, not following a simple Mendelian monogenic manner. Individuals with dark skin color show an increased prevalence of keloids with an estimated incidence of 4%–16%, 15 times higher than in Caucasian populations. NEDD4 was proven to be a candidate gene in Chinese Han and Japanese populations (Fujita et al., 2019). Epigenetic modifications are composed of DNA methylations, histone modifications, and non-coding RNA regulations. Non-coding RNAs are mainly composed of three types, microRNAs—miRNAs, long non-coding RNAs—lncRNAs, and circular RNAs—circRNAs. Emerging research showed that epigenetics plays a crucial role in the molecular pathogenesis of keloids. Epigenetic modification is considered to be an important regulator in the initial and sustained activation of keloid fibroblasts (Lv et al., 2020).

## CircRNA expression profiles in keloids

High-throughput sequencing and gene microarray have demonstrated aberrant expression profiles of circRNAs in keloid tissue and fibroblasts (Table 1). The altered expression profiles of circRNAs in keloid tissue may contribute to the etiology and pathophysiology of keloids by impacting signaling pathways relevant to the scaring process.

**TABLE1:** Expression and function of dysfunctional circRNA in keloids.

CircRNAs	Expression	Functional	Possible mechanism	Ref
**CircCOL5A1**	Up	Promoted HKF proliferation, migration, invasion, and ECM production. Inhibited HKF apoptosis	miR-877-5p/EGR1	24, 25
**CircSLC8A1**	Down	Inhibited HKF proliferation, migration, and ECM deposition. Promoted HKF apoptosis	miR-181a-5p/HIF1AN	34
**circ_0043688**	Up	Promoted HKF proliferation, migration, and invasion. Inhibited HKF apoptosis	miR-145-5p/FGF2	26
**CircPDE7B**	Up	Promoted HKF proliferation, migration, and invasion. Inhibited HKF apoptosis	miR-661/FGF2	27
**circ_101,238**	Up	Promoted HKF proliferation. Inhibited HKF apoptosis	miR-138-5p/CDK6	28
**Circ_0008450**	Up	Inhibited human keratinized epithelial cell proliferation, migration, and EMT process. Promoted HKF apoptosis	Runx3 tTGF-β/Smad	31
**circNRIP1**	Up	Promoted HKF proliferation and ECM production. Inhibited HKF apoptosis	FXR1-mediated upregulation of miR-503-3p and miR-503-5p	32
**circPTPN12**	Down	Inhibited HKF proliferation, migration, and invasion. Promoted HKF apoptosis	miR-21-5p/Wnt	35
**circ_0057452**	Up	Promoted HKF viability, proliferation, and migration. Inhibited HKF apoptosis	miR-1225-3p/AF4/FMR2	33

In a high-throughput sequencing research, Zhang et al. identified 411 differentially expressed (DE) circRNAs performed in three HKFs and normal dermal fibroblasts, with 206 circRNAs upregulated and 205 circRNAs downregulated. Bioinformatics analyses showed that 411 DE circRNAs mainly participated in cell apoptosis and focal adhesion processes, as well as PI3K-Akt, Rap1, and metabolic signaling pathways (Zhang et al., 2020a). Shi et al. used a circRNA microarray assay to determine circRNA expression in keloid tissue compared with paired normal skin tissue. They showed that 52 circRNAs were upregulated and 24 downregulated in keloids (Shi et al., 2020). In addition, further analysis found that circRNAs could interact with miR-29a, miR-23a-5p, and miR-1976 (Shi et al., 2020). Wang et al. performed high-throughput sequencing research in keloid tissue compared with normal skin tissue. Among 154 DE circRNAs, 81 circRNAs were upregulated and 73 circRNAs were downregulated (Wang et al., 2019). Li et al. performed high-throughput sequencing and showed that circRNAs might act as ceRNAs in the development of human hypertrophic scars (Li et al., 2018). Pang et al. performed microarray technology in four patient-derived keloid dermal fibroblasts (KDFs) compared with normal dermal fibroblasts (NDFs) (Pang et al., 2022). They detected a total of 327 DE circRNAs, with 195 upregulated and 132 downregulated circRNAs. The DE circRNAs were mainly enriched in cell function of the cytoskeleton, tight junctions, axonal guidance, and morphogenesis of the epithelium.

## Mechanisms of circRNAs in keloids

Myofibroblasts derived from quiescent resident skin fibroblasts are the principal cell type responsible for extracellular matrix (ECM) accumulation. The imbalance between fibroblast proliferation and apoptosis is the cytological basis for the continuous proliferation of keloids, and it highlights the epigenetic contribution to keloid formation by modulating the balance between fibroblast proliferation and apoptosis.

### Keloid-promoting circRNAs

Lv et al. showed that circCOL5A1 was upregulated in keloid tissues and HKFs. Silencing of circCOL5A1 inhibited HKF proliferation, migration, and ECM deposition and promoted the rate of apoptosis. Moreover, circCOL5A1 sponges miR-7-5p to release Epac1 through the PI3K/Akt signaling pathway (Lv et al., 2021). Similarly, Jiao et al. showed that circCOL5A1 expression is obviously higher in keloid tissues and HKFs. CircCOL5A1 knockdown hindered HKF proliferation, invasion migration, and ECM deposition, while promoting the rate of cell apoptosis. Moreover, circCOL5A1 could upregulate the expression level of EGR1 *via* sponging miR-877–5p (Jiao et al., 2022). Liu et al. found increased hsa_circ_0043688 and FGF2 and decreased miR-145-5p in human keloid samples and HKFs using RT-qPCR. Functional analysis showed that silencing of hsa_circ_0043688 repressed HKF proliferation, invasion, and migration and promoted apoptosis. Collectively, hsa_circ_0043688 modulated keloid progression *via* miR-145-5p/FGF2 (Liu et al., 2022a). CircPDE7B was highly expressed in keloid samples and HKFs. High circPDE7B accelerates HKF proliferation, migration, and invasion and hindered the rate of apoptosis. Moreover, circPDE7B functioned as a ceRNA for miR-661. The circPDE7B/miR-661/FGF2 ceRNA regulatory axis plays crucial roles in the pathogenesis of keloids (Wu et al., 2022). YANG et al. showed that circ_101,238 was significantly increased in keloid samples. Circ_101,238 was proven to sponge miR-138-5p, with CDK6 as a target. Transfection with sh-circ_101,238 inhibited HKF proliferation, while promoting apoptosis *via* regulating the miR-138-5p/CDK6 pathway (Yang et al., 2020). Runt-related transcription factors (Runx) play critical roles in the development and cancers (Date and Ito, 2020). Knockdown of circ_0008450 may reduce cell proliferation, migration, and EMT process of human keratinized epithelial cells and promoted apoptosis through increasing Runx3 and repressing the TGF-β/Smad signal pathway (Chen et al., 2020). The TGF-β/Smad signaling pathway is the most crucial pathway involved in the excessive production of collagen in the fibroblasts and myofibroblasts (Zhang et al., 2020b). WANG et al. demonstrated circNRIP1 was higher in keloid tissue than in adjacent skin tissue. Absence of circNRIP1 inhibited the proliferation and ECM-associated protein production while increasing apoptosis in HKFs. CircNRIP1 maintained FXR1 stability by inhibiting ubiquitination and degradation of FXR1, which increased the expression of miR-503-3p and miR-503-5p. In summary, circNRIP1 contributes to keloid development *via* FXR1-mediated upregulation of miR-503-3p and miR-503-5p (Wang et al., 2021). Gao et al. demonstrated that hsa_circ_0057452 and AFF4 are remarkably higher in keloids than in matched normal skin tissues. Hsa_circ_0057452 knockdown suppressed cell proliferation, viability, and migration, while accelerating the rate of apoptosis of HKFs. MiR-1225-3p is downregulated and showed a reverse effect on HKF function. Collectively, hsa_circ_0057452 regulates AFF4 and promotes keloid formation by sponging miR-1225-3p (Gao et al., 2022). Zhu et al. showed that circ_005745 induced keloid progression *via* upregulating GAB1 (Zhu et al., 2022).

### Keloid suppressor circRNAs

**FIGURE 1:**
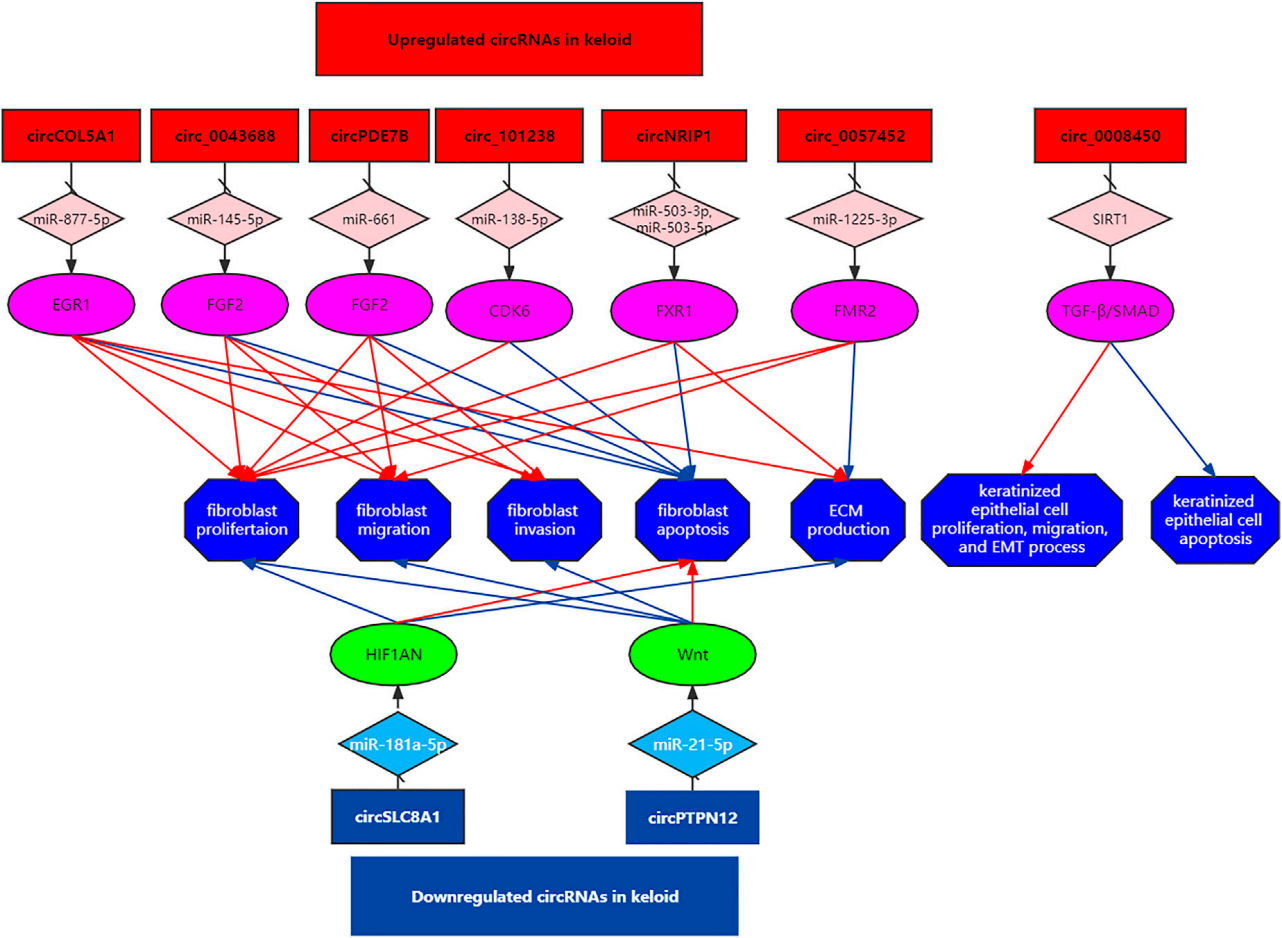
CircRNAs regulated gene expression *via* sponging miRNAs and played crucial roles in keloid development.

Yuan et al. showed that the level of circSLC8A1 declined in keloid tissues and HKFs. Overexpression of circSLC8A1 inhibited cell proliferation, migration, and ECM production and elevated cell apoptosis of HKFs. MiR-181a-5p is a direct sponging target of circSLC8A1, and HIF1AN was the downstream effect factor of miR-181a-5p. Taken together, circSLC8A1 inhibited keloid progression by regulating the miR-181a-5p/HIF1AN axis (Yuan et al., 2022). CircPTPN12 expression was downregulated in keloid tissue compared with the adjacent normal skin. Silencing of circPTPN12 accelerated HKF proliferation, migration, and invasion and suppressed apoptosis. CircPTPN12 could sponge miR-21-5p, while SMAD7 was the downstream effect factor of miR-21-5p. MiR-21-5p was a direct target of circPTPN12. In summary, silencing of circPTPN12 promotes keloid formation by activating the Wnt pathway sponging miR-21-5p (Liu et al., 2022b) (Figure 1 and Table 1).

## Conclusion and future perspective

Some circRNAs are profibrotic and their upregulation contributed to the development of keloids. Those profibrotic circRNAs include circCOL5A1、circ_0043688、circPDE7B、circ_101,238、circ_0008450、circNRIP1, and circ_0057452. However, some circRNAs are anti-fibrotic and their reduction inhibits the development of keloids. Those anti-fibrotic circRNAs include circSLC8A1 and circPTPN12. Gain- and loss-of-function studies have proven that deregulated circRNAs may regulate the processes underlying keloid formation and development.

CircRNAs are expected to be a potential diagnostic and therapeutic target in the management of keloids. For example, si-circCOL5A1 inhibited the growth and ECM deposition of keloids in the skin of nude mice (Lv et al., 2021). Further investigation into keloid-related circRNAs is needed to identify more effective prophylactic and clinical treatment strategies for keloids.
